# Molecular Determinants of Species-Specific Interactions Between Protein Kinase R and Poxvirus K3 Orthologs

**DOI:** 10.3390/v17121550

**Published:** 2025-11-26

**Authors:** Chorong Park, Greg Brennan, Chen Peng, Chi Zhang, Jingxin Cao, Loubna Tazi, Stefan Rothenburg

**Affiliations:** 1 Department of Medical Microbiology and Immunology, School of Medicine, University of California Davis, Davis, CA 95616, USA; chorong.park@northwestern.edu (C.P.); gregory.brennan@lmunet.edu (G.B.); chizhang@health.ucdavis.edu (C.Z.); ltazi@health.ucdavis.edu (L.T.); 2 College of Veterinary Medicine, China Agricultural University, Beijing 100107, China; pengchenea@cau.edu.cn; 3 Viral Diseases Division, National Microbiology Laboratory, Public Health Agency of Canada, Winnipeg, MB R3E 3R2, Canada; jingxin.cao@phac-aspc.gc.ca; 4 Department of Medical Microbiology, College of Medicine, University of Manitoba, Winnipeg, MB R3T 2N2, Canada

**Keywords:** poxviruses, sheeppox virus, goatpox virus, vaccinia virus, protein kinase R, K3L, translational regulation

## Abstract

Protein kinase R (PKR) is an antiviral protein that is involved in molecular “arms races” with viral antagonists. As a result, some PKR inhibitors, including the vaccinia virus (VACV) protein K3 and its orthologs from other poxviruses only inhibit PKRs of selected species. We previously reported contrasting inhibition patterns of human, sheep, and cow PKRs by VACV K3 and the sheeppox virus (SPPV) K3 ortholog, SPPV 011. Here we show that the differential sensitivities of cow and sheep PKRs to VACV K3 were mediated by only two residues in PKR helix αG. In contrast, SPPV 011 sensitivities were governed by additional residues and regions. Analysis of the PKR sensitivities from 20 mammalian species to VACV K3 and SPPV 011 revealed four different sensitivity patterns: some PKRs were inhibited by only one K3 ortholog, as previously reported, whereas other PKRs were either resistant or sensitive to both inhibitors. Furthermore, we characterized a residue (K45) in VACV K3 that is involved in the species-specific inhibition of PKR. Mutating this residue increased the inhibition of sheep but not human PKR, whereas it decreased the inhibition of mouse PKR, highlighting that a single mutation in a viral protein can result in distinct species-dependent inhibition changes.

## 1. Introduction

In order for viruses to cause productive infections, they have to overcome many challenges, including attaching to host cells via host receptor molecules, entry into the cells, appropriate intracellular trafficking, manipulation and harnessing of the host transcriptional, translational and metabolic machinery, evading host restriction factors, correct assembly of virus progeny, and egress from the cells. Failure to overcome any one of these barriers can lead to the failure of virus replication [1,2]. Recurrent virus–host interactions result in evolutionary pressure on both hosts and viruses and lead to molecular arms races [3]. Genetic changes as a result of these conflicts have been utilized to infer molecular evolutionary trajectories through, for example, the identification of amino acids evolving under positive selection in the interacting proteins [4].

Virus-generated RNAs can be sensed by pathogen recognition receptors, which often act as host restriction factors. Many of these restriction factors affect mRNA translation by either inhibiting the translation machinery directly or indirectly by modifying or degrading viral and host RNAs [5]. In turn, many viruses have evolved strategies to undermine the actions of these restriction factors [6]. One important host restriction factor that many viruses have to overcome to successfully replicate is protein kinase R (PKR), a pattern recognition receptor with broad antiviral activity. PKR consists of two N-terminal double-stranded (ds) RNA-binding domains (dsRBD) followed by a linker region and C-terminal kinase domain (KD), which allow it to act as both a sensor and an effector. PKR is activated by dsRNA, which is produced during the replication cycle of most viruses [7]. Recognition of dsRNA by the dsRBDs leads to PKR dimerization and subsequent autophosphorylation. The KD of activated PKR then phosphorylates the alpha subunit of the eukaryotic translation initiation factor 2 (eIF2) on serine 51, which leads to the inhibition of cap-dependent mRNA translation [8]. Viruses have evolved many different viral PKR antagonists, which target different steps during the PKR activation pathway including recognition of dsRNA, dimerization, auto-phosphorylation, as well as dephosphorylation of eIF2α [9,10].

Poxviruses are generally susceptible to the antiviral effects of PKR and have consequently evolved different PKR inhibitors [11,12,13,14]. The most prevalent PKR inhibitors in poxviruses are called K3 (encoded by K3L/OPG41) and E3 (encoded by E3L/OPG65) in vaccinia virus [15,16]. K3 contains an S1 domain, which is homologous to the one found in eIF2α, and acts as a pseudosubstrate inhibitor to prevent the interaction of PKR with eIF2α, thereby allowing mRNA translation to proceed [11,17]. E3 is a dsRNA-binding protein, which prevents both proper dsRNA-binding by PKR and PKR dimerization [12,18]. Both K3L and E3L were identified as host range genes, because their deletion affected only virus replication in a subset of cells. Initially, E3L was described to be dispensable for infection of Syrian hamster BHK-21 cells, while K3L was dispensable for the infection of human HeLa cells [19]. A molecular explanation for the host range function of K3L and E3L is that human PKR is mostly resistant to K3 inhibition and sensitive to E3 inhibition, whereas Syrian hamster PKR is sensitive to K3 inhibition and resistant to E3 inhibition [20]. Interestingly, PKR from closely related hamster species showed differential sensitivities to inhibition, with Armenian hamster PKR being resistant to K3 inhibition and sensitive to E3 inhibition, and Chinese hamster PKR being sensitive to both K3 and E3 inhibition [20]. The differential sensitivities of PKR to K3 and E3 inhibition are likely a result of the above-mentioned molecular arms races, which led to the rapid evolution of PKR genes with many sites being under positive selection during vertebrate evolution [20,21,22,23]. In addition, VACV K3 orthologs from other poxviruses were shown to inhibit PKR in a species-specific manner [23,24,25,26,27,28,29].

Capripoxviruses cause economically important diseases in livestock and comprise three virus species: sheeppox virus (SPPV), goatpox virus (GTPV), and lumpy skin disease virus (LSDV). SPPV and GTPV both infect sheep and goats with mortality rates approaching 50%, while LSDV causes 1–10% mortality rates in cattle [30]. We have previously shown that the SPPV and GTPV K3 orthologs 011 strongly inhibited sheep, goat, and human PKRs, but only weakly inhibited cattle and mouse PKRs. In contrast, VACV K3 only weakly inhibited sheep, goat, and human PKRs, but strongly inhibited cattle and mouse PKRs. This correlated with the replication of VACV strains that contained only VACV K3L, SPPV 011, or GTPV 011 as their sole PKR inhibitor in cells of sheep, goat, human, and cow origin [27].

In this study, we investigated the molecular basis for the contrasting inhibition profiles exerted by SPPV/GTPV 011 and VACV K3, by performing sub-domain exchanges between sheep and cow PKRs, complemented by site-directed mutagenesis, using luciferase-based reporter assays. We expanded the number of tested mammalian PKRs to 20 and discovered that the inhibition profiles of SPPV 011 and VACV K3 are not necessarily contrasting by showing that some PKRs were either sensitive or resistant to inhibition by both inhibitors. We finally analyzed the PKR inhibitory potential of a spontaneous K3L mutation (K45E) in VACV that was isolated from a sheep cell line and allowed VACV replication [31]. This mutation led to a better inhibition of sheep PKR, but not of human PKR, and actually led to decreased inhibition of mouse PKR, indicating that some mutations in K3 could provide replication advantages in some species, while having the opposing effect in another species.

## 2. Materials and Methods

### 2.1. Cell Line

HeLa PKR-knock-down (^kd^) cells (kindly provided by Dr. Charles Samuel) [32] and HeLa PKR-knock-out (^ko^) (kindly provided by Dr. Adam Geballe) [33] were maintained in Dulbecco’s Modified Eagle’s Medium (DMEM) supplemented with 5% fetal bovine serum and 1 µg/mL puromycin (Sigma, Kawasaki, Japan) and 100 IU penicillin/streptomycin (Gibco, Grand Island, NY, USA), and cultured in a humidified incubator at 37 °C with 5% CO_2_.

### 2.2. Plasmids

The following PKR genes were used in the study: human (*Homo sapiens*) PKR (NP_001129123.1); Bornean orangutan (*Pongo pygmaeus*) PKR (XP_054329302.1); white-cheeked gibbon (*Nomascus leucogenys*) PKR (EU733257.1); rhesus macaque (*Macaca mulatta*) PKR (EU733261.1); red-chested mustached tamarin (*Saguinus labiatus*) PKR (ACI31222.1); cow (*Bos taurus*) PKR (NP_835210.2); goat (*Capra hircus*) PKR (XM_005686488.2); sheep (*Ovis aries*) PKR (KT272868); pig (*Sus scrofa*) PKR (XP_020941521.1); Bactrian camel (*Camelus bactrianus*) PKR (XP_010965954.1); horse (*Equus caballus*) PKR (AAX47275.1); European rabbit (*Oryctolagus cuniculus*) PKR (KT272867); mouse (*Mus musculus*) PKR (AAH16422.1); rat (*Rattus norvegicus*) PKR (XM_008764426.1); Turkish hamster (*Mesocricetus brandti*) PKR (MG702602.1); Chinese hamster (*Cricetulus griseus*) PKR (KT272869); Armenian hamster (*Nothocricetulus migratorius*) PKR (MG702601.1); guinea pig (*Cavia porcellus*) PKR (KT272870); cat (*Felis catus*) PKR (XP_019683233.1); dog (*Canis lupus familiaris*) PKR (NP_001041600.1). Cloning of these PKR genes into the pSG5 expression vector (Stratagene, La Jolla, CA, USA) was described previously [20,21,24,27,28,29]. Orangutan PKR, gibbon PKR, macaque PKR, and tamarin PKR were originally kindly provided by Dr. Nels Elde [22].

The cloning of pSG5-SPPV-011 (NP_659587.1), pSG5-GTPV-011 (YP_001293205.1), and pSG5-VACV-K3L (YP_232916.1) were previously described [21,27]. Cow PKR, sheep PKR mutants, and VACV K3L-K45E, VACV K3L-K45N, and SPPV 011-N49K were generated by site-directed mutagenesis, using overlapping primers containing targeted nucleotide sequences. Whole plasmid amplifications were performed with Phusion High-Fidelity polymerase (NEB), according to the manufacturer’s protocol. DNA was treated with Dpn I (NEB) for 1 h at 37 °C then transformed into DH5a E. coli chemically competent cells (New England Biolabs, Ipswich, MA, USA). Primers used for mutagenesis are described in Table 1. Domain swapping mutants were constructed by Gibson assembly (New England Biolabs, Ipswich, MA, USA). The N-lobe of the kinase domain of cow PKR was inserted into sheep PKR with primers 5′-CATTTACAGCGTGAACGAAAGGTTATTCAAC-3′ and 5′-CCTTTATCACAATATTCCATCTGGATGAAAAG-3′. The C-lobe of the kinase domain of cow PKR was inserted into sheep PKR with primers 5′-CTTTTCATCCAAATGGAATATTGTGATAAAGGTACGTTG-3′ and 5′-CTCGAGTCACTAACATGTGTTTCGTTTCTTTTTTTC-3′.

All plasmids obtained from mini-preps (Qiagen, Germantown, MD, USA) were confirmed by Sanger sequencing (DNA Technologies Core, UC Davis, Davis, CA, USA). For transfections, plasmids retransformed and midi-preps were prepared using the Nucleobond Xtra Midi Endotoxin Free DNA preparation kit (Macherey-Nagel, Bethlehem, PA, USA).

### 2.3. Luciferase-Based Reporter (LBR) Assay

Luciferase assays were performed as described [34]. Briefly, 5 × 10^4^ HeLa PKR^kd^ cells were seeded onto each well of a 24-well plate approximately 16 h before the experiment. Cells were co-transfected with 200 ng of the indicated PKR plasmid or derived mutants, 200 ng of the indicated K3 orthologs, and 50 ng of firefly luciferase (Promega, Madison, WI, USA) using GenJet reagent (Signagen, Frederick, MD, USA) at a DNA to GenJet ratio of 1:2. Empty pSG5 vector (vector control) was used to maintain the DNA concentration where appropriate. Forty-eight hours post-transfection, cells were lysed with mammalian lysis buffer (Goldbio, St. Louis, MO, USA). Luciferase activities were determined after adding luciferin (Promega, Madison, WI, USA) according to the manufacturer’s recommendations using a GloMax luminometer (Promega, Madison, WI, USA). Experiments were conducted in triplicate for each of the three independent experiments. Relative luciferase activities were obtained after normalization to the vector control.

### 2.4. PKR Phylogeny

The amino acid sequences of PKR from 42 different mammalian species were aligned using MUSCLE (v3.8) [35]. Ambiguously aligned positions were eliminated using Gblocks v0.91b [36]. The phylogenetic analysis was performed using the maximum likelihood approach, as implemented in PhyML v3.0 [37]. Branch support was estimated using the aLRT statistics in PhyML v3.0. The phylogenetic tree was constructed using the default substitution model, as implemented in the automatic model selection in PhyML [38]. One hundred bootstrap replicates were performed to estimate the nodal support at branch points. The resulting phylogenetic tree was visualized using FigTree v1.4.3.

### 2.5. Estimation of Positively Selected Sites in Ruminant PKRs

To determine amino acid sites under positive selection, we first performed a multiple sequence alignment using nucleotide sequences of 20 ruminant species (Table 2) in MUSCLE [35]. Correct codons were manually adjusted using MacClade (Sinauer Associates, Sunderland, MA, USA). A codon-based analysis was performed using the program codeml, as implemented in the PAML v4.8 software package [39]. Selection pressure was inferred by estimating the ratio of non-synonymous to synonymous substitutions (*ω* = dN/dS) across codons. Two pairs of models were used: M1a (nearly neutral) vs. M2a (positive selection), and M7 (beta) vs. M8 (beta and *ω*). Model likelihoods were estimated to determine the best-fit model using a Likelihood Ratio Test (LRT). Positive selection sites (pP > 0.95) were identified using the Bayesian empirical Bayes (BEB) approach [40].

## 3. Results

### 3.1. Species-Specific PKR Inhibition by VACV, SPPV, and GTPV K3 Orthologs

**Figure 1:**
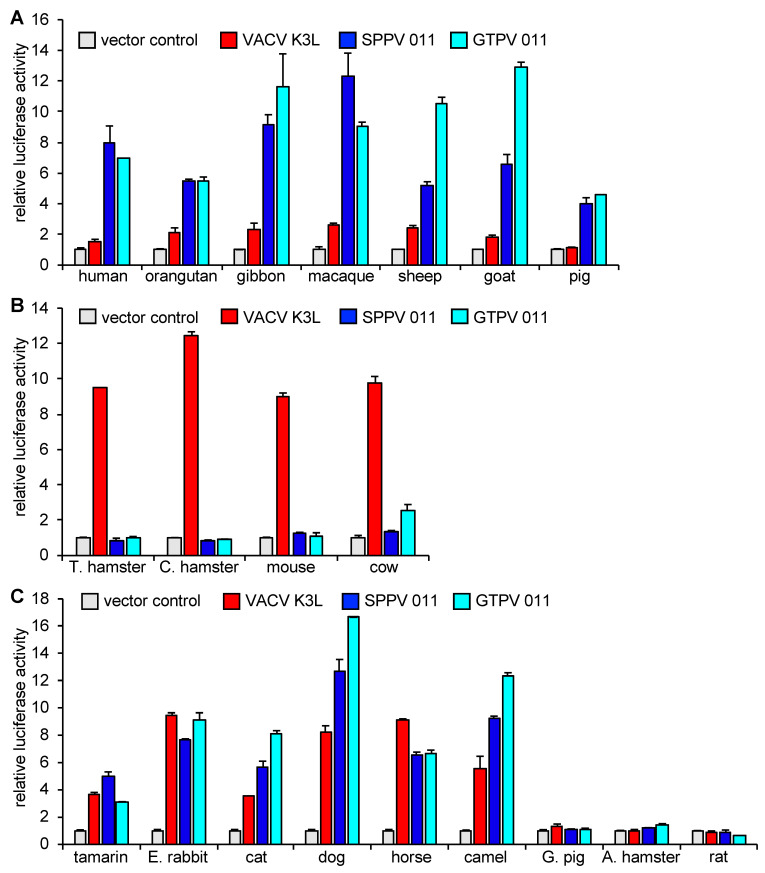
Differential sensitivities of PKRs from multiple species to VACV K3, and SPPV and GTPV 011. HeLa-PKR^kd^ cells were transfected with expression vectors encoding firefly luciferase (0.05 μg), PKR from indicated species (0.2 μg), and 0.2 μg of VACV K3L or SPPV and GTPV 011 plasmids. Luciferase activities were measured 48 h after transfection and normalized to PKR-only transfected cells to obtain relative luciferase activities. Error bars represent the standard deviations from three independent transfections. Results shown are representative of three independent experiments. (**A**) Results for PKRs that were resistant or mostly resistant (≤3-fold inhibition) to VACV K3, and sensitive to SPPV and GTPV 011 (≥3-fold inhibition). (**B**) Results for PKRs that were sensitive to VACV K3, and resistant to SPPV and GTPV 011. (**C**) Results for PKRs that were either sensitive or resistant to VACV K3L, SPPV 011, and GTPV 011.

**Figure 2:**
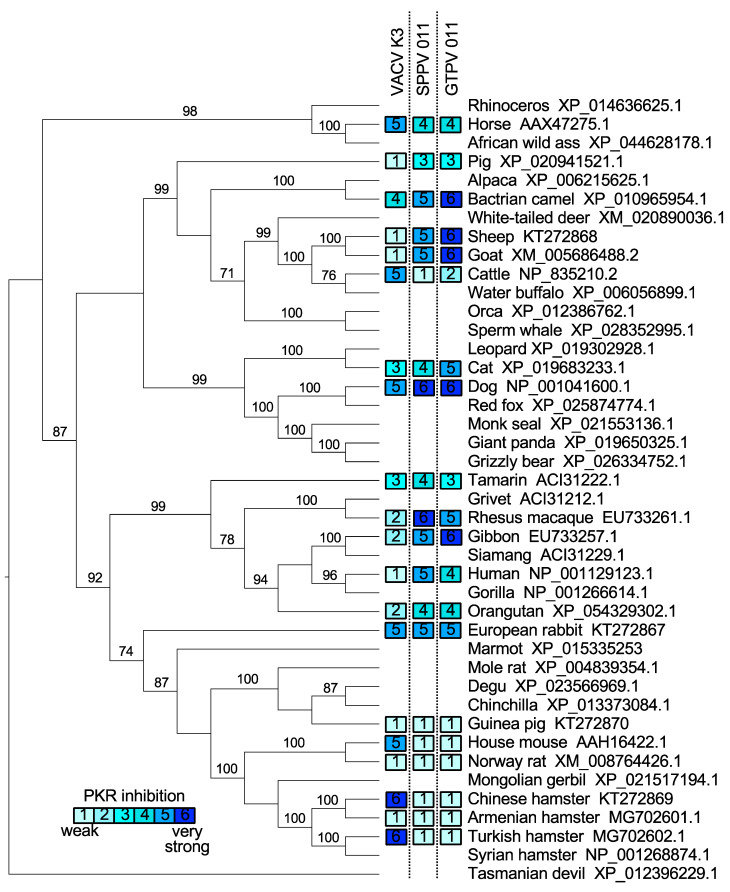
Sensitivities of tested PKRs to VACV K3 and SPPV and GTPV 011 projected on a PKR phylogenetic tree. A maximum likelihood phylogenetic tree of PKRs from the indicated mammalian species including the ones tested in this study was generated. The tree was rooted to Tasmanian devil PKR. Bootstrap values ≥ 70 are indicated on top of the branches. Relative sensitivities of PKRs to VACV K3, SPPV 011, or GTPV 011 obtained from multiple experiments with PKR:K3 ortholog are shown on a scale from 1 to 6: 1 = no or very weak inhibition (≤2-fold); 2 = weak inhibition (2- to 3-fold); 3 = intermediate low inhibition (3- to 5-fold); 4 = intermediate high inhibition (5- to 7-fold); 5 = high inhibition (7- to 10-fold); 6 = very high inhibition (≥10-fold).

Our previous findings showed that cow and mouse PKRs were sensitive to VACV K3 inhibition, and resistant to SPPV and GTPV 011, whereas human, goat, and sheep PKRs were largely resistant to VACV K3 inhibition, while being sensitive to SPPV and GTPV 011 inhibition [27]. VACV K3 shares only 36% amino acid identity with the SPPV and GTPV K3 orthologs, while the amino acid identity between SPPV 011 and GTPV 011 is 93%. This previously observed dichotomy of inhibition patterns indicated that efficient PKR inhibition by either K3 or capripoxvirus 011 might preclude inhibition by the other. In order to test whether this conclusion holds up for other PKRs, we extended this analysis to test the sensitivity of PKRs from 20 mammalian species to VACV K3, SPPV 011, and GTPV 011 in an established luciferase-based reporter (LBR) assay [21,34]. In this assay, PKR-deficient or PKR-depleted HeLa cells are co-transfected with a luciferase reporter plasmid, PKR, and PKR inhibitors or control plasmids. PKR is activated by dsRNA formed by overlapping transcripts generated from the transfected plasmids [41], and inhibits the translation of luciferase mRNA. Luciferase activity thus serves as a proxy for relative translation efficiency and is inversely correlated with PKR activity. The transfection of all tested PKRs resulted in the strong and comparable inhibition of luciferase activity (Supplementary Figure S1). PKRs from 11 out of the 20 tested species showed the two previously observed PKR inhibition patterns by the K3 orthologs. We found that PKRs derived from human, sheep, goat, orangutan, gibbon, macaque, and pig were largely resistant to VACV K3 (<3-fold inhibition) but sensitive to SPPV and GTPV 011 (>3-fold) inhibition (Figure 1A). In contrast, PKR derived from mouse, cow, Turkish hamster, and Chinese hamster were sensitive to VACV K3 inhibition but largely resistant to SPPV and GTPV 011 (Figure 1B). Notably, PKRs from the other species did not fit into this pattern. One group of PKRs, which comprised PKRs from tamarin, European rabbit, cat, dog, horse, and camel, were sensitive to both VACV K3 and SPPV/GTPV 011, whereas PKRs from Armenian hamster, guinea pig, and rat were resistant to inhibition by all tested K3 orthologs (Figure 1C). We also projected the relative sensitivities of the tested PKRs to VACV, SPPV, and GTPV K3 orthologs on a phylogenetic tree that was generated with PKRs from 42 species using a previously described susceptibility scale ranging from weakly sensitive to highly sensitive on a scale from 1 to 6, respectively [29] (Figure 2). These results show that the sensitivities to the tested K3 orthologs are not necessarily inversely coupled, and that the relatedness of PKRs was not a good predictor for the sensitivities of PKRs to inhibition by the K3 orthologs.

### 3.2. Differential Inhibition of Cow and Sheep PKRs Is Mediated by the C-Terminal Lobe of the Kinase Domain

**Figure 3:**
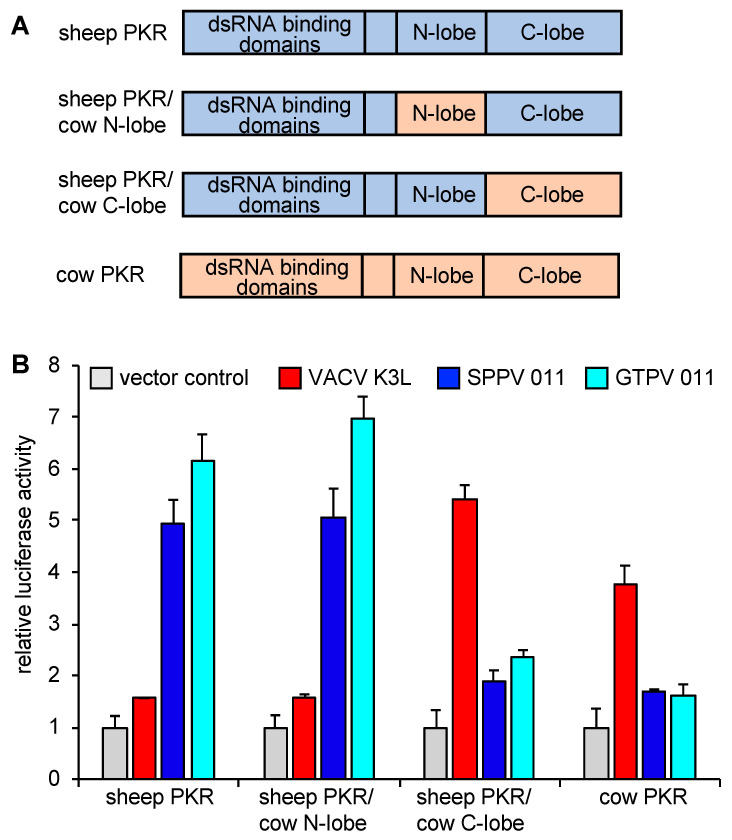
Identification of sub-domains of sheep and cow PKRs that are responsible for the differential sensitivities to VACV K3 and SPPV and GTPV 011. (**A**) Schematic representation of PKR domains. The kinase domain was divided into N- and C-lobes. Sheep and cow PKR domains are shown in violet or salmon colors, respectively. (**B**) HeLa-PKR^kd^ cells were transfected with expression vectors encoding firefly luciferase (0.05 μg), sheep, cow, or chimeric PKRs and 0.2 μg of VACV K3L, SPPV, or GTPV 011 (0.2 μg) plasmids. Luciferase activities were measured 48 h after transfection and normalized to PKR-only transfected cells to obtain relative luciferase activities. Error bars represent the standard deviations from three independent transfections. The results shown are representative of three independent experiments.

We next wanted to elucidate molecular determinants for the differential and opposing sensitivity patterns of sheep and cow PKRs to inhibition by VACV K3 and SPPV and GTPV 011. Because K3 orthologs interfere with the interaction between the kinase domain of PKR and eIF2α by structurally mimicking the S1 domain of eIF2α [17], we focused on the kinase domain of sheep PKR to identify the regions responsible for the differential PKR sensitivities to K3 orthologs. We first created chimeric sheep PKRs in which either the N-terminal or C-terminal lobes were replaced with the respective ones from cow PKR (Figure 3A). We compared sensitivities of these mutants, as well as those of sheep and cow PKR to inhibition by SPPV, GTPV, and VACV K3 orthologs in the LBR assay. Sheep and cow PKRs showed the previously observed sensitivities (Figure 3B). Sheep PKR containing the N-terminal lobe from cow PKR showed a comparable sensitivity to wild type sheep PKR, while sheep PKR containing the C-terminal lobe from cow PKR exhibited a sensitivity comparable to cow PKR, demonstrating that the C-terminal lobe defines the sensitivities to the tested K3 orthologs. The tested PKRs and mutants showed comparable activities in the LBR assay (Supplementary Figure S2A).

### 3.3. Helix αG Determines the Sensitivity of Cow PKR to VACV and Capripoxvirus K3 Inhibition

**Figure 4:**
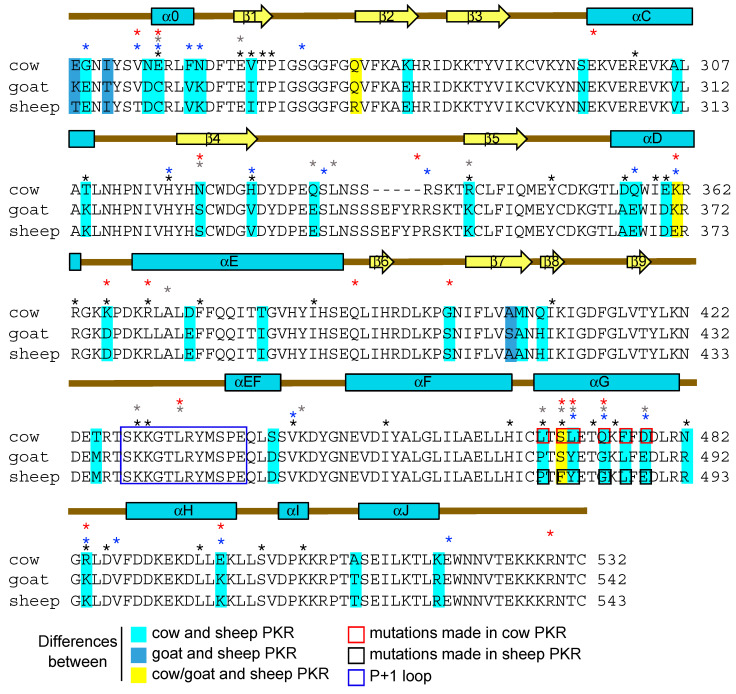
Multiple sequence alignment of the cow, goat, and sheep PKR kinase domains. Cow, goat, and sheep PKRs were aligned using Clustal Omega v1.2.4 (EMBL–EBI). Numbers indicate amino acid positions. Amino acid differences between the sequences are shaded in the indicated colors. The position of point mutations made in this study are indicated with red (cow) and black (sheep) boxes in the alignment. The secondary structure elements are based on the structure of human PKR [42]. The positions of residues under positive selection in datasets of vertebrate (black asterisks [21]), primate (blue asterisks [22]), rodents (gray asterisks [20]), or ruminant (red asterisks, this study) PKR sequences are indicated.

To identify the molecular determinants of the contrasting sensitivities between cow and sheep PKRs to VACV and capripoxvirus K3 orthologs, we performed a PAML analysis for positively selected residues using 20 available sequences for ruminant PKRs (Table 3). A total of 25 amino acid positions were identified as being under positive selection, 17 of which reside in the kinase domain (Table 3, Figure 4). A total of 12 of these sites in the kinase domain were previously identified to be under positive selection in datasets containing vertebrate, and/or primate, and/or rodent PKR sequences [20,21,22]. These analyses revealed a cluster of positively selected residues in helix αG with six amino acid differences between cow and sheep PKR sequences, and five differences between cow and goat PKRs (Figure 4). Because we previously observed similar sensitivity patterns between sheep and goat PKRs to viral inhibitors in the LBR assay, we focused our mutagenesis analyses on the differences in helix αG between cow and sheep PKRs.

**Figure 5:**
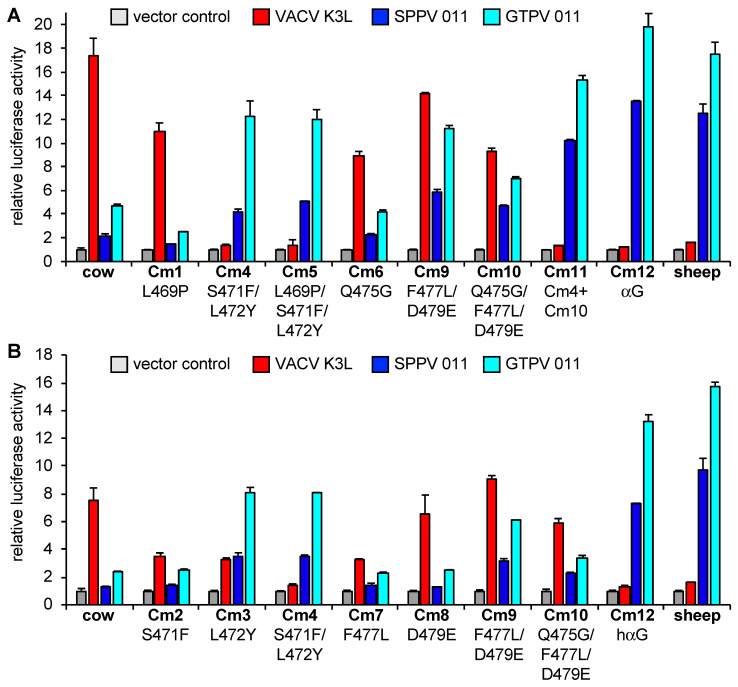
(**A**,**B**) Identification of residues that govern sensitivity of cow PKR to K3 orthologs. HeLa-PKR^kd^ cells were co-transfected with expression vectors encoding firefly luciferase (0.05 μg), cow PKR, or cow PKR mutants (Cm) (0.2 μg), and VACV K3L or SPPV and GTPV 011 (0.2 μg) plasmids as indicated. Mutations in PKRs are shown under the mutant names and are detailed in Table 4. Luciferase activities were measured 48 h after transfection and normalized to PKR-only transfected cells to obtain relative luciferase activities. Error bars represent the standard deviations from two independent transfections. The results shown are representative of three independent experiments.

We first generated mutants of cow PKR in which we replaced divergent residues alone and in different combinations with corresponding amino acids found in sheep PKR and designated them cow mutants (Cm) 1 through 12. All tested PKRs and PKR mutants exhibited comparable activities in the LBR assay (Supplementary Figure S2B). We next characterized their sensitivities to inhibition by K3 orthologs from VACV, SPPV, and GTPV in the LBR assay (Figure 5). For easier presentation, we also graded the relative inhibition on a previously developed susceptibility scale ranging from resistant or weakly sensitive to highly sensitive on a scale from 1 to 6, respectively (Table 4) [29]. The mutant Cm4 (S471F/L472Y) was resistant to VACV K3. Mutation of either S471F (Cm2) or L472Y (Cm3) resulted in partial resistance to VACV K3. Resistance to VACV K3 was also observed in all mutants (Cm4, Cm5, Cm11, Cm12) containing both S471F and L472Y mutations along with other mutations. In contrast, S471F (Cm2) and L472Y (Cm3) mutations alone only partially affected the sensitivity of cow PKR to SPPV and GTPV 011, and mutations of several additional residues in helix αG (C11 and Cm12) were necessary for a complete conversion to sheep PKR-like sensitivity. Cm10 (Q475G/F477L/D479E) showed increased sensitivity to SPPV and GTPV 011 inhibition, indicating that these residues contribute to the sensitivity to capripoxvirus 011 but not to VACV K3.

### 3.4. Regions Outside Helix αG in Sheep PKR Contribute to Its Sensitivity to SPPV and GTPV 011

**Figure 6:**
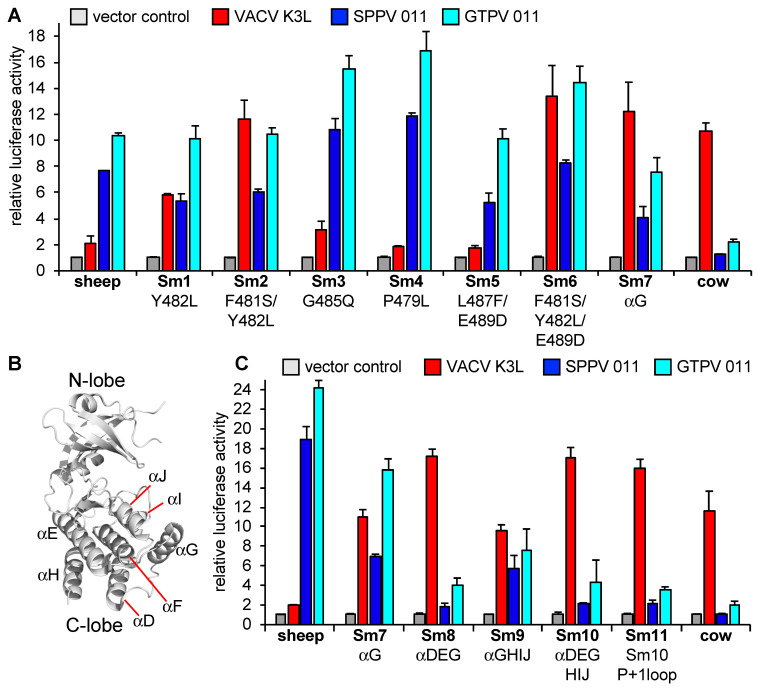
Identification of residues that govern sensitivity of sheep PKR to K3 orthologs. HeLa-PKR^kd^ cells were co-transfected with expression vectors encoding firefly luciferase (0.05 μg), sheep PKR, or sheep PKR mutants (Sm) (0.2 μg), and VACV K3L or SPPV and GTPV 011 (0.2 μg) plasmids as indicated. Mutations in PKRs are shown under the mutant names and are detailed in Table 4. Luciferase activities were measured 48 h after transfection and normalized to PKR-only transfected cells to obtain relative luciferase activities. Error bars represent the standard deviations from two independent transfections. The results shown are representative of at least three independent experiments. (**A**) Results are for helix αG mutants are shown. (**B**) Structure of the kinase domain of human PKR (Protein Data Bank ID code 2A1A) [42]. The secondary structures in C-lobe are indicated and used for the mutagenesis. (**C**) Results for sheep PKR mutants containing the indicated parts from cow PKR.

We next used the LBR assay to determine whether helix αG of sheep PKR is the sole determinant of sensitivity to VACV, SPPV, and GTPV K3 orthologs. Multiple sheep PKR constructs were generated using site-directed mutagenesis and sub-domain swapping and designated Sm1–Sm11 (Table 4). Similarly to the results obtained for cow PKR, mutations of F481S and Y482L (Sm2) in sheep PKR, which correspond to residues 471 and 472 in cow PKR, made sheep PKR sensitive to inhibition by VACV K3 (Figure 6). In line with this result, sheep PKR mutants Sm6 (F481S/Y482L/E489D) and Sm7 (helix αG), which contain the same substitutions (F481S, Y482L) as in Sm2 in addition to others, showed comparable increased sensitivity to VACV K3. Interestingly, we did not identify any mutations in helix αG of sheep PKR that made it resistant to SPPV and GTPV K3 orthologs. Instead, we observed slightly enhanced PKR sensitivities with mutations G485Q (Sm3) and P479L (Sm4) (Figure 6). These results suggest that in sheep PKR, unlike in cow PKR, regions outside of helix αG also contribute to the sensitivity to capripoxvirus K3 orthologs.

We further dissected the C-lobe of sheep PKR to narrow down the region(s) that influence the sensitivity of sheep PKR to inhibition by the capripoxvirus K3 orthologs. The PKR C-lobe comprises two paired antiparallel β strands and eight a helices (αD to αJ) [42] (Figure 4 and Figure 6B). Using this secondary structure annotation, we generated different sheep PKR chimeras that contain partial sequences from cow PKR. As we observed above, replacing helix αG in the sheep PKR with the counterpart of the cow PKR was sufficient to alter the sensitivity to VACV K3 but had only a minor impact on the sensitivity to SPPV and GTPV 011 (Figure 6C). Sm8, which contains cow PKR helices αD, αE, and αG phenocopied the sensitivity to inhibition. Similarly, Sm10 and Sm11, which also contain helices αD, αE, and αG in addition to other regions, showed comparable sensitivities. These results indicate that helices αD and/or αE contribute to the sensitivity of sheep PKR to capripoxvirus 011 inhibition.

### 3.5. Species-Specific Modulation of PKR Inhibition by VACV K3 Mutants

**Figure 7:**
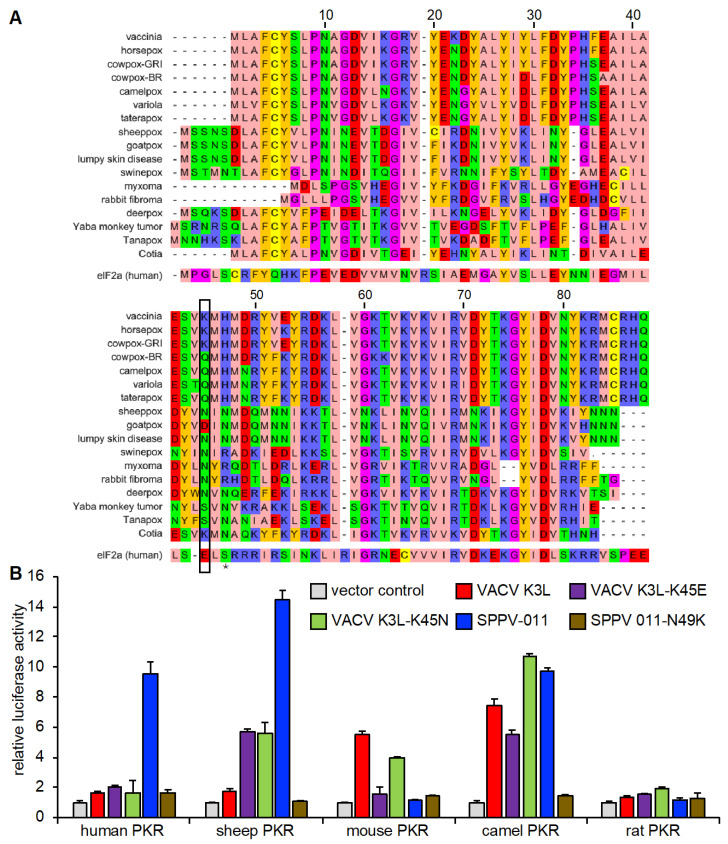
Characterization of VACV K3 mutants with altered species-specific PKR inhibition. (**A**) Multiple sequence alignment of selected poxvirus K3 orthologs and human eIF2α obtained with MUSCLE in MegAlignPro. Numbers indicate amino acid positions of VACV K3. Residues corresponding to K45 in VACV K3 are boxed. The asterisk indicates the phosphorylation site in eIF2α. (**B**) HeLa-PKR^kd^ cells were transfected with expression vectors encoding firefly luciferase (0.05 μg), PKR from indicated species (0.2 μg), and 0.2 μg of VACV K3L, SPPV 011, or their mutant plasmids. Luciferase activities were measured 48 h after transfection and normalized to PKR-only transfected cells to obtain relative luciferase activities. Error bars represent the standard deviations from three independent transfections. Results shown are representative of three independent experiments.

Consistent with the resistance of sheep PKR to VACV K3, it was previously reported that VACV lacking E3L was unable to replicate in sheep OA3 cells [31]. However, the authors identified a spontaneous variation in VACV K3L that led to the substitution of lysine 45 to glutamic acid (K45E), which enabled VACV replication in OA3 cells. SPPV 011 contains an asparagine (N) at the corresponding position (Figure 7A). In order to test the effect of this residue in VACV and SPPV K3 orthologs, we constructed VACV K3-K45E and VACV K3-K45N, as well as SPPV 011-N49K. We tested the ability of these mutants to inhibit PKRs from the four sensitivity groups: human and sheep (group 1), mouse (group 2), camel (group 3), and rat (group 4) in the LBR assay (Figure 7B). VACV K3-K45E showed increased inhibition of sheep PKR, consistent with the previous report [31]; however, inhibition levels did not reach that of SPPV 011. K3-K45E showed no increased inhibition of human, camel, and rat PKRs, and resulted in a substantially decreased inhibition of mouse PKR. VACV K3-K45N showed a comparable inhibition of sheep PKR as VACV K3 K45E, but only slightly decreased the inhibition of mouse PKR. Strikingly, the SPPV 011 N49K mutant completely lost its ability to inhibit human, sheep, mouse, and camel PKRs.

## 4. Discussion

An often-overlooked aspect of virus host range is the host species-specific inhibition of the innate immune response by viruses. Inhibition of the PKR pathway by viral antagonists represents a great model for studying the molecular basis for the underlying species-specific interactions. These investigations were started with two studies that uncovered strong signatures of positive selection in PKR genes from 20 primate or 27 vertebrate species, and revealed that PKR could be inhibited in a species-specific manner by vaccinia virus K3 [21,22]. Site-directed mutagenesis identified helix αG as a main contributor to the species-specific phenotype, but also showed that other sub-domains such as helices αD and αE contributed to this phenomenon. The species-specific inhibition of PKR was subsequently reported for other poxviruses, including leporipoxviruses, capripoxviruses, a vespertilionpoxvirus, and yatapoxviruses [23,24,25,26,27,28,29]. We previously found striking differences in the sensitivity of cow, sheep, goat, human, and mouse PKRs to K3 orthologs from capripoxviruses and VACV [27]. We focused on sheep and cow PKRs to decipher the molecular determinants of the contrasting PKR sensitivities to K3 orthologs from the two different poxvirus genera. Consistent with earlier studies, we found that the C-lobe of the PKR kinase domain was responsible for the differential inhibition. A more detailed mutational analysis revealed that swapping both residues in cow PKR (S471F and L472Y) and the mutation of the corresponding residues in sheep PKR (F481S, Y482L) reversed sensitivities to VACV K3, making cow PKR resistant and sheep PKR sensitive. We also identified these sites as being under positive selective pressure in ruminants and other species. Surprisingly, the swapping of both residues in either cow or sheep PKRs only partially changed the sensitivity to SPPV 011. In order to make cow PKR fully susceptible to SPPV 011 inhibition, additional substitutions in helix αG were required. In contrast, swapping the complete helix αG of sheep PKR with the cow PKR counterpart did only partially reduce the sensitivity to SPPV 011 inhibition, as it required additional mutations in helix αD and/or αE. While the exact additional residues were not resolved in this study, it shows that different parts of PKR can contribute to its sensitivity to the same inhibitors from different species. Based on the co-crystal structure of PKR with eIF2α, it seems unlikely that helices αD and αE directly interact with K3 orthologs. Instead, they might alter the positioning of helix αG as previously suggested [21,22,42]. The finding that only two residues in the helix αG dictated the sensitivities of cow and sheep PKRs to VACV K3, whereas more residues determined the sensitivities of sheep and cow PKRs to SPPV 011, shows that the sensitivity of PKR to different orthologous viral inhibitors can be partially determined by different amino acid positions.

While earlier results indicated that the sensitivities to VACV and capripoxvirus K3 orthologs might be inversely coupled, because cow and mouse PKRs were sensitive to VACV but resistant to SPPV and GTPV 011, whereas sheep, goat, and human PKRs were resistant to VACV K3 and sensitive to SPPV and GTPV 011, broadening the analysis to include PKRs from 15 additional species revealed that this was not the case, as we discovered that some PKRs were sensitive to all three tested inhibitors, whereas some PKRs were resistant to all. Among the resistant PKRs, pig and guinea PKRs were previously shown to be resistant to three additional orthopoxvirus K3 orthologs from variola, cowpox, and camelpox virus, whereas rat PKR was resistant to the variola virus K3 ortholog, but intermediately sensitive to cowpox and camelpox virus K3 orthologs [29]. While SPPV and GTPV 011 are very similar and share 96% amino acid identity with one another, and generally displayed similar PKR inhibition patterns, some smaller differences were detected. GTPV 011 showed somewhat stronger inhibition of PKR from some species than SPPV 011, including sheep, goat, and cat, whereas comparable inhibition (human, orangutan, horse) or stronger inhibition (macaque) by SPPV 011 was observed. Although the biological relevance of these smaller differences is currently unclear, it indicates that even those closely related viral proteins can cause differential PKR inhibition.

Overall, thirteen of the tested species’ PKRs were sensitive to SPPV and GTPV 011, while seven were resistant. It is noteworthy that most tested PKRs come from species that are not naturally infected by SPPV and GTPV. However, these species can be infected by other viruses, including poxviruses that contain K3 orthologs. It is possible that other extant or ancient viruses have exerted positive selective pressure on these PKRs and that, by chance, some PKRs were sensitive to SPPV and GTPV 011. This notion is supported by a previous study that showed that residues in helix αG of primate PKRs can not only modify sensitivities to VACV K3, but also to unrelated cytomegalovirus inhibitors [33,43].

The majority of research determining the molecular basis for the species-specific interaction of PKR has focused on the PKR side, with only limited detailed studies on the viral inhibitors. In a random mutagenesis, yeast-based screen VACV K3-H47R was identified as a better inhibitor of human PKR [44]. This residue corresponds to S51 in eIF2α which is phosphorylated by eIF2α kinases. VACV K3-H47R was also later discovered in VACV screen after serial passaging in a human HeLa cells, which conferred a replication advantage [45]. Hyperactivating mutations were also identified in a yeast-based mutagenesis screen of the myxoma virus K3 ortholog M156. In this study, a loss-of-function mutation was also identified, which might have helped the myxoma virus to become partially attenuated in Australia [24]. The SPPV 011-K45E was previously identified as allowing VACV replication in sheep cells [31]. Glutamic acid is also found in human eIF2α at the corresponding position, whereas sheeppox possesses an asparagine. Consistent with the previous report, K3-K45E showed stronger inhibition of sheep PKR, although inhibition levels did not reach that of SPPV 011. However, this mutation did not increase the inhibition of human PKR, and even resulted in the weakened inhibition of mouse PKR, indicating that beneficial mutations might increase viral replication in one species, but could have decreased replication in another species. Introduction of asparagine at this position (K3-K45N), which is found in sheeppox 011, resulted in a comparable sheep PKR inhibition as K45E, but only slightly affected the inhibition of mouse PKR. Residue 45 in VACV K3 is located between helices α1 and α2 in VACV K3, and is in close proximity to H47, which corresponds to the phosphorylation site of eIF2α, and likely interacts directly with PKR [17,42].

Overall, our study provides novel insights into the molecular basis for host-specific inhibition of an important antiviral protein by viral antagonists by elucidating which amino acid residues in both PKR and K3 from different species influence species-specific interactions, and by discovering the different sensitivity patterns of PKRs from different species to three orthologous viral inhibitors.

## Figures and Tables

**Table 1:** Primers used for plasmid mutagenesis.

Primer Name	Mutation	Forward Primer Sequences (5′—3′) (Reverse Primers Are Reverse Complement of Forward Primers)	Template
Cm1	L469P	GAACTTCATATATGTCCCACTTCTTTAGAAACACAG	cow PKR
Cm2	S471F	TCATATATGTCTCACTTTTTTAGAAACACAGAAGTT	cow PKR
Cm3	L472Y	TATGTCTCACTTCTTACGAAACACAGAAGTTCTTTG	cow PKR
Cm4	S471F, L472Y	TTCATATATGTCTCACTTTTTACGAAACACAGAAGTTCTTT	cow PKR
Cm5	L469P, S471F, L472Y	CTTCTTCATATATGTCCCACTTTTTACGAAACACAG	cow PKR
Cm6	Q475G	TCTCACTTCTTTAGAAACAGGGAAGTTCTTTGACGAT	cow PKR
Cm7	F477L	CTTTAGAAACACAGAAGTTATTTGACGATCTAAGGA	cow PKR
Cm8	D479E	TAGAAACACAGAAGTTCTTTGAAGATCTAAGGAATGGC	cow PKR
Cm9	F477L, D479E	TAGAAACACAGAAGTTATTTGAAGATCTAAGGAATGGC	cow PKR
Cm10	Q475G, F477L, D479E	ACTTCTTTAGAAACAGGGAAGTTATTTGAAGATCTA	cow PKR
Cm11	S471F, L472Y, Q475G, F477L, D479E	CATATATGTCTCACTTTTTACGAAACAGGGAAGTTATTT	cow PKR
Cm12	sheep helix αG	CTTCTTCATATATGTCCCACTTTTTACGAAACAGGG	cow PKR
Sm1	Y482L	CATATATGTCCCACTTTTTTAGAAACAGGGAAGTTATTTG	sheep PKR
Sm2	F481S, Y482L	CTTCATATATGTCCCACTAGTTTAGAAACAGGGAAGTT	sheep PKR
Sm3	G485Q	CCCACTTTTTACGAAACACAGAAGTTATTTGAAGATC	sheep PKR
Sm4	P479L	GAACTTCTTCATATATGTCTCACTTTTTACGAAACAGG	sheep PKR
Sm5	L487F, E489D	CGAAACAGGGAAGTTCTTTGATGATCTAAGGAGAGGC	sheep PKR
Sm6	F481S, Y482L, E489D	CGAAACAGGGAAGTTCTTTGATGATCTAAGGAGAGGC	sm2
Sm7	cow helix αG	CTAGTTTAGAAACACAGAAGTTCTTTGATGATCTAAGGAGAGGC	sheep PKR
Sm8	cow helix αD + E + G	GGAGTGCATTATATACATTCAGAACAGTTAATTCAC	sheep PKR
Sm9	cow helix αG + H+ I + J	GCAGAACTTCTTCATATATGTCTCACTTCTTTAGAAAC	sheep PKR
Sm10	Sm9 + cow helix αD+ E	GGAGTGCATTATATACATTCAGAACAGTTAATTCAC	Sm9
Sm11	Sm10 + p + 1loop	GGAGTGCATTATATACATTCAGAACAGTTAATTCAC	Sm10
VACV K3L-K45E	K45E	GCTATCTTGGCAGAGAGTGTTGAACTGCATATGGATAGAT	VACV K3L
VACV K3L-K49E	K49N	GCTATCTTGGCAGAGAGTGTTGAGATGCATATGGATAGAT	VACV K3L
SPPV 011-N49K	N49K	TTGTAATAGATTATGTTGAAATAAACATGGATCAA	SPPV 011

**Table 2:** Genes from *Ruminantia* included in the positive selection analysis.

Species	Common Name	Accession	Family	Subfamily
*Capra hircus*	goat	XM_018055123.1	*Bovidae*	*Caprinae*
*Capricornis sumatraensis*	Sumatran serow	XP_068829084.1	*Bovidae*	*Caprinae*
*Ovibos moschatus*	muskox	KAL1288457.1	*Bovidae*	*Caprinae*
*Budorcas taxicolor*	takin	XP_052505461.1	*Bovidae*	*Caprinae*
*Ovis aries*	sheep	XP_004007349.1	*Bovidae*	*Caprinae*
*Ovis canadensis*	bighorn sheep	XP_069439646	*Bovidae*	*Caprinae*
*Oryx dammah*	scimitar-horned oryx	XP_040080570.1	*Bovidae*	*Hippotraginae*
*Bos taurus*	cattle	XM_005212570.5	*Bovidae*	*Bovinae*
*Bison bison bison*	American bison	XP_010836908.1	*Bovidae*	*Bovinae*
*Bos mutus*	wild yak	XP_070235833.1	*Bovidae*	*Bovinae*
*Bos javanicus*	banteng	XM_061432077.1	*Bovidae*	*Bovinae*
*Bubalus kerabau*	carabao	XP_055395787.1	*Bovidae*	*Bovinae*
*Bubalus bubalis*	water buffalo	XP_044781760.1	*Bovidae*	*Bovinae*
*Odocoileus virginianus*	white-tailed deer	XP_020745697.2	*Cervidae*	*Capreolinae*
*Rangifer tarandus platyrhynchus*	Svalbard reindeer	CAI9174154.1	*Cervidae*	*Capreolinae*
*Cervus canadensis*	elk	XP_043324268.1	*Cervidae*	*Cervinae*
*Cervus elaphus*	red deer	XP_043774162.1	*Cervidae*	*Cervinae*
*Dama dama*	European fallow deer	XP_061011579	*Cervidae*	*Cervinae*
*Muntiacus reevesi*	Reeves’ muntjac	XP_065785502.1	*Cervidae*	*Muntiacinae*
*Moschus berezovskii*	dwarf musk deer	XP_055255352.1	*Moschidae*	

**Table 3:** Positively selected sites in ruminant PKRs.

Residue	Posterior Probabilities
(Goat PKR)	(pP)
54 K	0.984 *
74 I	0.961 *
95 R	0.992 **
128 R	0.976 *
182 A	0.964 *
225 V	0.965 *
247 A	0.998 **
250 V	0.961 *
259 V	0.990 *
261 C	1.000 **
303 E	0.987 *
325 S	0.956 *
346 R	0.984 *
371 K	0.957 *
376 D	0.975 *
380 L	0.987 *
400 Q	0.963 *
409 S	0.971 *
443 L	0.981 *
480 S	0.988 *
481 Y	0.991 **
484 G	0.993 **
494 K	0.978 *
507 K	0.952 *
539 R	0.971 *

* indicates pP ≥ 0.95; ** indicates pP ≥ 0.99.

**Table 4:** Mutants of cow and sheep PKRs and their relative sensitivities to inhibition by VACV K3 and SPPV 011.

PKR/		Relative Inhibition by	
PKR Mutant	Mutations	VACV K3	SPPV 011
Cow PKR	wild type	6	1
Cm1	L469P	6	1
Cm2	S471F	3	1
Cm3	L472Y	3	3
Cm4	S471F, L472Y	1	3
Cm5	L469P, S471F, L472Y	1	4
Cm6	Q475G	5	2
Cm7	F477L	3	1
Cm8	D479E	5	1
Cm9	F477L, D479E	6	4
Cm10	Q475G, F477L, D479E	5	3
Cm11	Cm4 + Cm10	1	6
Cm12	Helix αG	1	6
Sheep PKR	wild type	1	6
Sm1	Y482L	4	4
Sm2	F481S, Y482L	6	4
Sm3	G485Q	3	6
Sm4	P479L	1	6
Sm5	L487F, E489D	1	4
Sm6	F481S, Y482L, E489D	6	5
Sm7	helix αG	6	4
Sm8	helices αD, αE, αG	6	1
Sm9	helices αG, αH, αI, αJ	6	4
Sm10	helices αD, αE, αG, αH, αI, αJ	6	2
Sm11	Sm10 + P + 1 loop	6	2

## Data Availability

All data necessary for the interpretation of the findings presented in this work are contained within the manuscript figures.

## Supplementary Materials

The following supporting information can be downloaded at: https://www.mdpi.com/article/10.3390/v17121550/s1, Figure S1: Activities of analyzed mammalian PKRs. Figure S2: Activities of analyzed PKR mutants.

## Abbreviations

The following abbreviations are used in this manuscript:

PKR	Protein kinase R
eIF2	Eukaryotic translation initiation factor 2
VACV	Vaccinia virus
SPPV	Sheeppox virus
GTPV	Goatpox virus
ds	Double-stranded
LBR	Luciferase-based reporter
