# Supplementary material for: Molecular Determinants of Species-Specific Interactions Between Protein Kinase R and Poxvirus K3 Orthologs
Source: Viruses. 2025 Nov 26;17(12):1550. doi: 10.3390/v17121550 (PMC12737409; doi:10.3390/v17121550)
Supplement: Supplementary file 1 [file viruses-17-01550-s001.zip › viruses-3973134-supplementary.pdf]

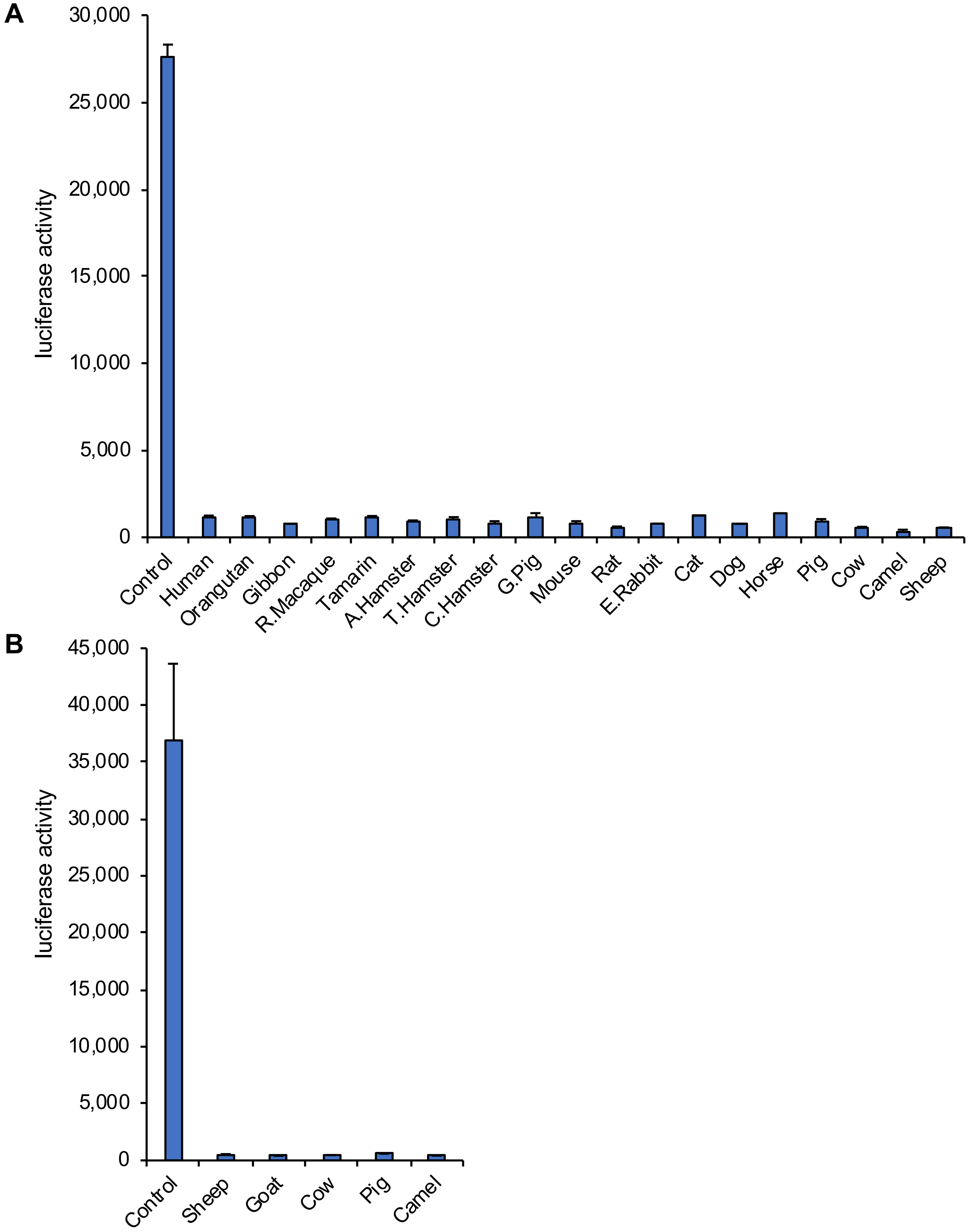

Supplementary Figure S1. Activities of analyzed mammalian PKRs. HeLa-PKR^{kd} cells were transfected with expression vectors encoding firefly luciferase (0.05 µg), and 0.2 µg empty SG5 plasmid (control), or PKR from indicated species (0.2 µg). Total luciferase activities were obtained 48 hours after transfection. Error bars represent the standard deviations from three independent transfections.

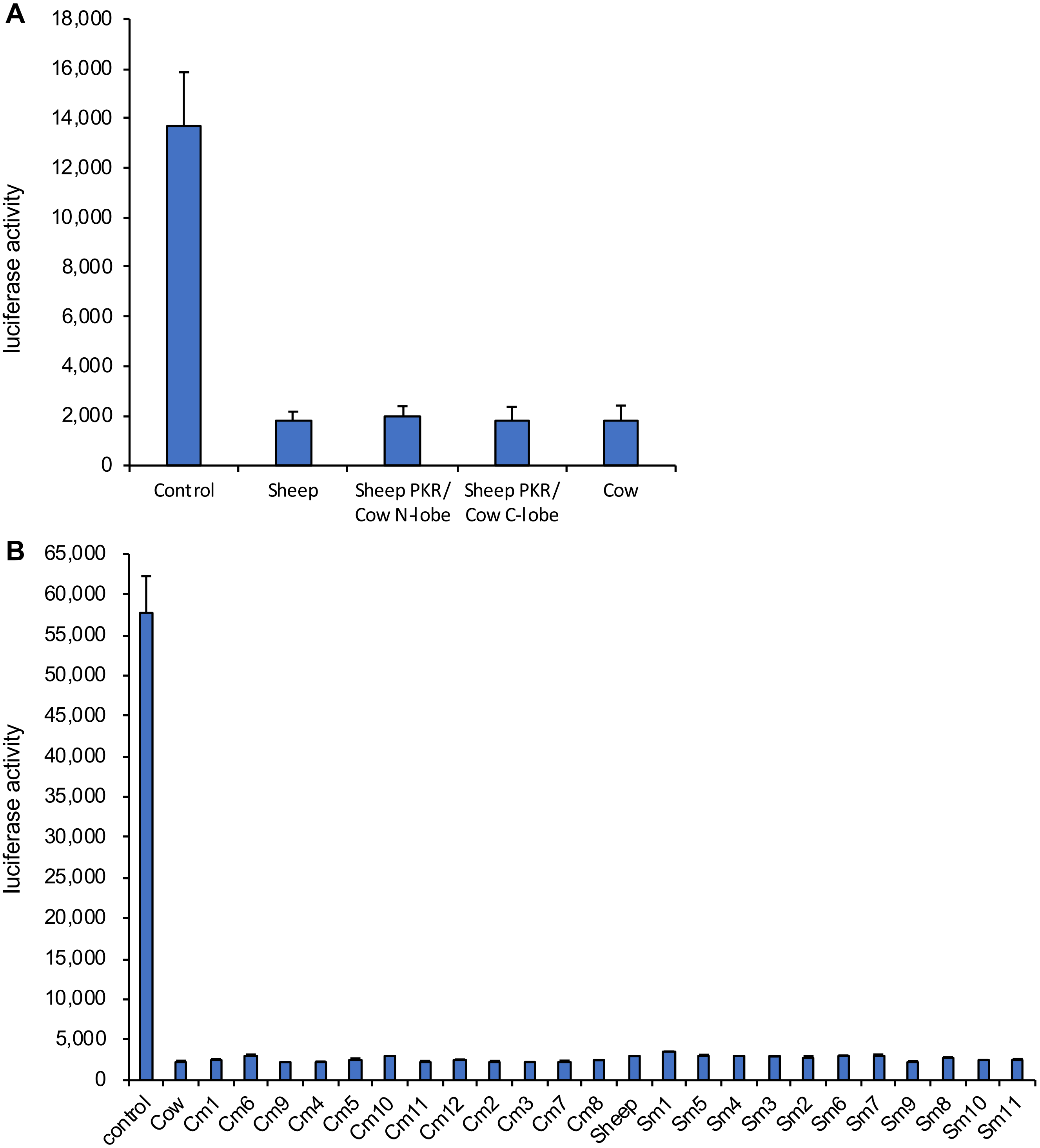

Supplementary Figure S2. Activities of analyzed PKR mutants. HeLa-PKR^{ko} cells were transfected with expression vectors encoding firefly luciferase (0.05 µg), and 0.2 µg empty SG5 plasmid (control), or 0.2 µg PKR from indicated species, or indicated PKR mutants. Total luciferase activities were obtained 48 hours after transfection. Error bars represent the standard deviations from three independent transfections.
